# Perceived fear of COVID-19 among Nepali older adults

**DOI:** 10.1093/geroni/igab046.3215

**Published:** 2021-12-17

**Authors:** Saruna Ghimire, Uday Narayan Yadav, Om Prakash Yadav, Janardan Subedi

**Affiliations:** 1 Miami University, Oxford, Ohio, United States; 2 University of New South Wales, SYDNEY, New South Wales, Australia; 3 Ministry of Health and Population, Kathmandu, Bagmati, Nepal

## Abstract

Although coronavirus-disease-2019 (COVID-19) impacted everyone in some ways, it disproportionally impacted the older population. Given their increased vulnerability to severe illness and mortality, the ongoing pandemic has created greater distress, anxiety, and fear among the older population. In Nepal, a South Asian country nestled in the Himalayas between India and China, most stories of older adults are untold – both in the pre-COVID-19 and the COVID-19 era. This study aimed to explore the perceived fear of COVID-19 among Nepali older adults. A cross-sectional study was conducted between July-September 2020 among 847 older adults (≥60years) residing in three districts of eastern Nepal. The seven-item Fear of COVID-19 Scale assessed the perceived fear of COVID-19; higher scores on the scale (ranges 7 to 35 indicated greater fear. A sizeable proportion of the participants' reported being afraid (35%), anxious (32%), uncomfortable (24%), clammy (14%), and sleepless (12%), while 28% were fearful of losing their life due to COVID-19. In adjusted regression analysis, older age group, Dalit (minority) ethnicity, and remoteness to the health facility were associated with greater fear of COVID-19. Surprisingly, pre-existing health conditions were inversely associated with fear of COVID-19. Greater fear of COVID-19 amidst the pandemic, although anticipated, urges us to reflect on the most vulnerable groups' psychological needs not just during COVID-19 but in the future events of pandemics and public health emergencies. Fear during emergencies could be battled with accurate and effective information as well as better preparedness and psychosocial interventions.

